# Supplementary material for: Identifying pre-existing conditions and multimorbidity patterns associated with in-hospital mortality in patients with COVID-19
Source: Sci Rep. 2022 Oct 15;12:17313. doi: 10.1038/s41598-022-20176-w (PMC9568958; doi:10.1038/s41598-022-20176-w)
Supplement: Supplementary file 1 — Supplementary Information. [file 41598_2022_20176_MOESM1_ESM.pdf]

Identifying pre-existing conditions and multimorbidity patterns associated with in-hospital mortality in patients with COVID-19

Magda Bucholc, PhD[1,2], Declan Bradley, PhD[2,3], Damien Bennett, PhD[2], Lynsey Patterson, PhD[2], Rachel Spiers, MPH[2], David Gibson, PhD[4], Hugo Van Woerden PhD[2,5,6], Anthony J Bjourson, PhD[4]

[1] Intelligent Systems Research Centre, School of Computing, Engineering & Intelligent Systems, Ulster University, Londonderry, BT48 7JL, United Kingdom

[2] Public Health Agency, Belfast, BT2 8BS, United Kingdom

[3] Centre for Public Health, Queen's University Belfast, Belfast, BT12 6BA, United Kingdom

[4] Northern Ireland Centre for Stratified Medicine, Biomedical Sciences Research Institute, Ulster University, Londonderry, BT47 6SB, United Kingdom

[5] Institute of Nursing and Health Research, Ulster University, Coleraine, BT52 1SA 3, United Kingdom

[6] Division of Rural Health and Wellbeing, University of the Highlands and Islands, Inverness, IV2 3JH, United Kingdom

Corresponding author: Magda Bucholc, PhD; Intelligent Systems Research Centre, School of Computing, Engineering & Intelligent Systems, Ulster University, Londonderry, BT48 7JL, United Kingdom. E-mail: m.bucholc@ulster.ac.uk

Abstract

We investigated the association between a wide range of comorbidities and COVID-19 in-hospital mortality and assessed the influence of multimorbidity on the risk of COVID-19-related death using a large, regional cohort of 6036 hospitalized patients. This retrospective cohort study was conducted using Patient Administration System Admissions and Discharges data. The International Classification of Diseases 10th edition (ICD-10) diagnosis codes were used to identify common comorbidities and the outcome measure. Individuals with lymphoma (odds ratio [OR], 2.78;95%CI,1.64-4.74), metastatic cancer (OR, 2.17; 95%CI,1.25-3.77), solid tumour without metastasis (OR, 1.67; 95%CI,1.16-2.41), liver disease (OR: 2.50, 95%CI,1.53-4.07), congestive heart failure (OR, 1.69; 95%CI,1.32-2.15), chronic obstructive pulmonary disease (OR, 1.43; 95%CI,1.18-1.72), obesity (OR, 5.28; 95%CI,2.92-9.52), renal disease (OR, 1.81; 95%CI,1.51-2.19), and dementia (OR, 1.44; 95%CI,1.17-1.76) were at increased risk of COVID-19 mortality. Asthma was associated with a lower risk of death compared to non-asthma controls (OR, 0.60; 95%CI,0.42-0.86). Individuals with two (OR, 1.79; 95%CI, 1.47-2.20; $P < 0.001$), and three or more comorbidities (OR, 1.80; 95%CI, 1.43-2.27; $P < 0.001$) were at increasingly higher risk of death when compared to those with no underlying conditions. Furthermore, multimorbidity patterns were analysed by identifying clusters of conditions in hospitalised COVID-19 patients using k-mode clustering, an unsupervised machine learning technique. Six patient clusters were identified, with recognisable co-occurrences of COVID-19 with different combinations of diseases, namely, cardiovascular (100%) and renal (15.6%) diseases in patient Cluster 1; mental and neurological disorders (100%) with metabolic and endocrine diseases (19.3%) in patient Cluster 2; respiratory (100%) and cardiovascular (15.0%) diseases in patient Cluster 3, cancer (5.9%) with genitourinary (9.0%) as well as metabolic and endocrine diseases (9.6%) in patient Cluster 4; metabolic and endocrine diseases (100%) and cardiovascular diseases (69.1%) in patient Cluster 5; mental and neurological disorders (100%) with cardiovascular diseases (100%) in patient Cluster 6. The highest mortality of 29.4% was reported in Cluster 6.

Introduction

The coronavirus disease 2019, caused by severe acute respiratory syndrome coronavirus-2 (SARS-CoV-2), continues to pose a major threat to public health worldwide. The clinical spectrum of COVID-19 ranges from an asymptomatic state or mild respiratory symptoms to severe viral pneumonia and acute respiratory distress syndrome[1,2]. Although severe illness from COVID-19 can occur in healthy individuals of any age, the risk of severe symptoms with rapid disease progression and death is significantly higher in older adults, with people over the age of 80 having 20-fold increased risk of COVID-19-related death than adults aged 50–59 years[3]. The severity of, and mortality due to, COVID-19 are higher in males than females[4]. The OpenSAFELY analysis of 17,278,392 primary care records, including 10,926 COVID-19 deaths, showed that older people, males, and those living in deprived areas were at significantly higher risk of COVID-19-related death[3]. Furthermore, various pre-existing conditions, such as, cardiovascular disease[4-8], diabetes[4,5,7], hypertension[4,7,8], respiratory diseases[4,7,8], cancer[4,9], chronic kidney disease[5,10], obesity[5,11,12] have been associated with increased risk of COVID-19 occurrence, the development of severe health conditions, and higher risk of death. In fact, reports showed that over 90% of individuals that died as a direct consequence of SARS-CoV-2 infection had at least one pre-existing condition[13]. Chen et al.[14] reported that patients with severe or critical COVID-19 who died were on average 17 years older, more likely to be male, and more likely to have a comorbidity such as hypertension, diabetes, cardiovascular disease, or chronic lung disease. A study by the Chinese Center for Disease Control and Prevention (44,672 confirmed cases, 1,023 deaths) showed that several pre-existing medical conditions including cardiovascular disease, hypertension, diabetes, respiratory disease, and cancer, substantially increased the risk of COVID-19 adverse outcomes[15]. In the UK, a cross-sectional survey of 16,749 individuals hospitalized with COVID-19 demonstrated that patients with chronic cardiac, pulmonary, and kidney disease, cancer, dementia and obesity were at higher risk of death[16]. People with obesity who

contracted SARS-CoV-2, not only required invasive mechanical ventilation more often but were also 48% more likely to die[17,18].

Given the evidence suggesting that COVID-19 disproportionately impacts the elderly and people living with long-term conditions, more research into the factors associated with deaths due to COVID-19 involving different population groups and settings is needed to better inform the design of strategies to reduce harm to those at highest risk. Furthermore, there is a paucity of research on the impact of multimorbidity on the risk of a fatal outcome associated with COVID-19. In this study, we aimed to estimate the impact of multiple long-term conditions on COVID-19 in-hospital mortality and assess the influence of multimorbidity on the risk of COVID-19-related death using a large, regional cohort of hospitalized patients.

Results

Analytical population

The dataset used in this study was based on the hospital identifying the admission as a COVID-19 admission and coding the method of admission accordingly. In effect, 8524 records identified as COVID-19 hospital admissions were initially considered for inclusion. Patients (n = 1075) were then excluded from the analysis if COVID-19 was not confirmed by laboratory testing or not diagnosed clinically or epidemiologically as reflected in the ICD-10 coding (note that the method of admission was not retrospectively amended). In addition, 552 patients with no ICD-10 codes were excluded. In effect, analyses were restricted to patients with ICD-10 diagnosis code for COVID-19, including primary or secondary diagnosis code of U07.1 and U07.2, with complete ICD-10 data on pre-existing health conditions. The ICD-10 diagnosis codes from multiple records of hospital admissions for a specific individual, including transfers between or within hospitals, were combined and the duplicate ICD-10 codes removed (n = 861). This resulted in a subset of 6036 (70.8%) hospitalized COVID-19 cases included in the analysis (Fig. 1). Out of 6036 patients, 5955 (98.7%) represented cases when COVID-19 was confirmed by laboratory testing (ICD-10 diagnosis code U07.1) while 81 (1.3%) corresponded

to COVID-19 cases that were diagnosed clinically or epidemiologically since laboratory testing was inconclusive or not available (ICD-10 diagnosis code U07.2).

Demographic and clinical characteristics of COVID-19 patients are shown in Table 1. The median (interquartile range, IQR) age of the 6036 patients was 72 (57-82) years. Patients that died were older (80 (73-86) years) than those that survived hospitalization (68 (54-80) years). Of those admitted to hospital with COVID-19, there was a higher proportion of males (3213, 53.2%) than females (2823, 46.8%), with men having a higher risk of COVID-19–associated mortality (711, 58%) compared to women (514, 42%) ($P < 0.001$). While 1713 patients (28.4%) had no previous comorbidities, 2010 patients (33.3%) had one, 1507 patients (25.0%) had two, and 806 patients (13.3%) had three or more comorbidities, with hypertension (22.7%), chronic obstructive pulmonary disease (19.8%), and diabetes (15.6%) being the most common conditions.

Comorbidities and in-hospital mortality in patients with COVID-19

An overview of associations between different comorbidities examined in this study and in-hospital mortality in patients with COVID-19 are shown in Table 2. Only 15.3% (187/1225) of patients who died in hospital from COVID-19 had no documented long-term conditions. Out of patients who were discharged, 31.7% (1526/4811) had no record of pre-existing long-term conditions. However, it is worth noting that patients with no record of pre-existing long-term conditions who died were significantly older (median (IQR): 81 (72-86) years) than those who were discharged (58 (44-72) years) ($p < 0.05$). Age was the factor most strongly associated with higher risk of COVID-19–associated mortality with a clear large gradient seen with increasing age (e.g., OR, 3.62; 95%CI, 1.83-7.71; $P < 0.001$ in patients aged 50-59 years; OR, 8.98; 95%CI, 4.69-17.24; $P < 0.001$ in patients aged 60-69 years; and OR, 14.53; 95%CI, 7.62-27.69; $P < 0.001$ in patients 70-79 years compared with those aged 40 to 49 years). The largest mortality risk was observed in patients aged ≥ 80 years compared with those aged 40-49 years (OR, 23.77; 95%CI, 12.48-45.27; $P < 0.001$). No significant difference in risk of COVID-19-related death was found between the age groups 0-40 years and 40-49 years (OR,

0.60; 95%CI, 0.20-1.77, p = 0.36). The risk of death was significantly higher in men (OR, 1.41; 95%CI, 1.22-1.62; *P* < 0.001). Furthermore, individuals with obesity (OR, 5.28; 95%CI, 2.92-9.52; *P* < 0.001), lymphoma (OR, 2.78; 95%CI, 1.64-4.74; *P* < 0.001), metastatic cancer (OR, 2.17; 95%CI, 1.25-3.77; *P* = 0.006), solid tumour without metastasis (OR, 1.67; 95%CI, 1.16-2.41; *P* = 0.006), liver disease (OR: 2.50, 95%CI, 1.53-4.07; *P* < 0.001), congestive heart failure (OR, 1.69; 95%CI, 1.32-2.15; *P* < 0.001), chronic obstructive pulmonary disease (OR, 1.43; 95%CI, 1.18-1.72; *P* < 0.001), renal disease (OR, 1.81; 95%CI, 1.51-2.19; *P* < 0.001), dementia (OR, 1.44; 95%CI, 1.17-1.76; *P* < 0.001) were at increased risk of in-hospital mortality. People with asthma were significantly less likely to die due to COVID-19 (OR, 0.60; 95%CI, 0.42-0.86; *P* = 0.005). No significant associations were observed between cardiac arrhythmias, diabetes, hypertension, hypothyroidism, rheumatoid arthritis/collagen vascular diseases, depression, neurological disorders, and risk of death (Table 2).

Increasing number of comorbidities was associated with a greater COVID-19 in-hospital mortality risk. Individuals with one pre-existing health condition had an adjusted OR for in-hospital death of 1.44 (95%CI, 1.18-1.75; P < 0.001), compared to those with no pre-existing health conditions (Table 3). Furthermore, the risk of death was increasingly higher in patients with two (OR, 1.79; 95%CI, 1.47-2.20; P < 0.001), and three or more comorbidities (OR, 1.80; 95%CI, 1.43-2.27; P < 0.001). Analyses stratified by sex showed a similar trend, i.e., both men and women with multiple pre-existing health conditions had increased odds of death when compared to those with no pre-existing health conditions (Table 3). However, the interaction test showed that the effect of the number of pre-existing conditions on the risk of COVID-19-related death did not differ significantly between males and females.

Multimorbidity clusters

Out of 6,036 hospitalised patients, 2313 (38.3%) had ≥ 2 conditions. The optimal k-modes clustering solution yielded 6 patient groups. The number of patients in each group ranged from 648 (Cluster 2) to 2293 (Cluster 3). The identified patient clusters had significant differences in mortality, ranging from 15.3% (Cluster 4) to 29.4% (Cluster 6). Median (IQR) age ranged

from 63 (49-78) (Cluster 4) to 82 (74-86) (Cluster 6). Table 4 describes the prevalence of diseases in each patient cluster. Within each patient cluster, disease groups with the highest prevalence were highlighted in bold while other less common conditions with a higher prevalence than in other clusters were underlined. Cluster 1 was characterised by cardiovascular (100.0%), genitourinary (15.6%), and respiratory diseases (9.9%). Cluster 2 included a high percentage of patients with mental and neurological disorders (100%), metabolic and endocrine diseases (19.3%), and genitourinary diseases (9.4%). It was also Cluster 2 that had the highest percentage of patients with digestive diseases (2.5%). Note that digestive disease group included only patients with liver disease. Cluster 3 was characterized by respiratory (100%), cardiovascular (15.0%), and metabolic and endocrine diseases (12.7%) while Cluster 5 corresponded to the patient group with a high prevalence of metabolic & endocrine (100%), cardiovascular (69.1%), and genitourinary diseases (15.0%). The highest percentage of patients with cancer (5.9%) was found in Cluster 5. Finally, Cluster 6, with the highest mortality, was characterized by cardiovascular diseases (100.0%), and mental and neurological disorders (100%). Other featured conditions in this group were respiratory diseases (15.7%). This cluster had also the highest median (IQR) age of 82 (74-86). Several conditions were present in multiple patient clusters; however, their prevalence significantly differ.

Sensitivity analysis

The results of two sensitivity analyses, based on identifying COVID-19 related admissions with ICD-10 code diagnosis: 1) including only primary or secondary diagnosis code of U07.1 (Table 5) and 2) including U07.1 or U07.2 as the primary code (Table 6), were consistent with those of the primary analysis. The magnitude and significance of associations between potential risk factors and the outcome measure remained robust under different assumptions. The difference in results between the primary and sensitivity analyses was observed only for neurological disorders, i.e., neurological disorders were found to be associated with increased risk of COVID-19-related deaths in sensitivity analysis in which COVID-19 deaths were

defined as COVID-19 related admissions with ICD-10 diagnosis code U07.1 or U07.2 as the primary code for clinical diagnosis (OR, 1.45; 95%CI, 1.04-2.01; P = 0.03).

Compared with people with no underlying conditions, the risk of death was significantly higher for those with one or more comorbidities (OR, 1.63; 95%CI, 1.36-1.94; P < 0.001) (Table S2 in Supplementary Material). Moreover, the risk of death was increasingly higher in patients with two and three or more comorbidities (Table S3 and S4 in Supplementary Material). These results strengthened the credibility of our main findings by highlighting the significant association between underlying health conditions and in-hospital mortality in individuals with COVID-19.

Since the primary analysis indicated that asthma was associated with a decreased risk of COVID-19-related death, we also performed a third sensitivity analysis to compare the risk of death from COVID-19 in people with asthma compared to those with no documented comorbidities. Our results showed no difference in risk of death from COVID-19 in people with asthma compared to patients with no record of underlying long-term conditions (OR, 1.22; 95%CI, 0.86-1.75; P = 0.27), suggesting that the presence of other comorbidities may have an impact on the significance of results of the primary analysis. We intend to investigate the extents of multimorbidity in patients with asthma as well as the relationship between asthma medications and different multimorbidity patterns in future work.

Discussion

Early recognition of high-risk and critically ill patients has become a priority in improving treatment and reducing mortality among patients who contracted SARS-CoV-2[2]. In this study, we examined the impact of multiple long-term conditions on the in-hospital mortality in individuals with COVID-19 to determine risk factors for COVID-19-related deaths. Our findings can help improve the effectiveness of management of COVID-19 patients and contribute to further development of policies for prevention and response to COVID-19 and its critical outcomes. Using a large, well-documented, regional cohort of hospitalised COVID-19 patients,

we found that pre-existing health conditions, including obesity, liver disease, renal disease, metastatic cancer, solid tumour without metastasis, lymphoma, congestive heart failure, chronic obstructive pulmonary disease, and dementia are clinical risk factors associated with COVID-19 mortality, with chronic obstructive pulmonary disease, renal disease, and dementia being the most prevalent among those that died.

Our results are consistent with several studies[4,5,7,19-22]. Chronic obstructive pulmonary disease (COPD) was found to increase the odds of death by nearly 3-fold in a large meta-analysis of 30 studies that examined the vital status of COVID-19 patients with COPD[19]. Substantial mortality rates in COVID-19 patients with COPD were also observed by other studies[20,21]. It has been suggested that the association between COPD and risk of poor outcomes in COVID-19 might be related to the fact that the innate and acquired antiviral immune responses in individuals with COPD are impaired, leading to delayed virus clearance[19]. Dementia was identified as a major risk factor for death in COVID-19 cases[22,23]. Wang et al.[22] showed that the odds of COVID-19-related death in patients with dementia doubled when compared to individuals without dementia, with the highest mortality risk in adults with vascular dementia (OR, 3.17; 95% CI, 2.97–3.37, $P < 0.001$). Some evidence suggested that elevated risk of neurological complications from COVID-19 in people with dementia might be caused by the pre-existing brain pathology[24]. For example, the breakdown of the blood-brain barrier, i.e., a defence mechanism against disease-causing pathogens, in patients with Alzheimer's disease and vascular dementia, can increase the ability of bacterial, fungal, and viral pathogens to access the brain more easily and this in turn may have an effect on the severity of COVID-19 and associated fatal outcomes[25,26]. A number of studies investigated the impact of chronic diseases and health conditions on risk of COVID-19-related death through multivariate analyses[4,5,7-10,27]; however, some of them were based on a small sample size, included a limited list of underlying medical conditions or focused on the impact of a specific medical condition on COVID-19 mortality adjusted for demographic and/or socioeconomic characteristics[7,9,10]. Moreover, previous evidence on the relationship between multimorbidity

and COVID-19-related death was limited[4,5]. A UK prospective observational study of 20,133 patients who were hospitalised with COVID-19 showed that the risk of death was higher for patients with dementia, chronic pulmonary disease, kidney disease, cardiac disease, liver disease, malignancy, and obesity[5]. Another study based on 10,926 COVID-19-related deaths reported similar results: individuals with COVID-19 and underlying kidney disease, liver disease, cardiovascular disease, chronic respiratory diseases, obesity, and recent history of haematological malignancy or other cancers had a greater risk of dying[27]. Cancer, possibly due to its ability to cause immunodeficiency inherently or through medication, was identified as a major risk factor for COVID-19-related deaths in several studies[4,5,27]. In individuals with heart failure and kidney disease, both the SARS-CoV-2 infection and the immune response to the viral infection could destabilize pre-existing conditions, leading to the development of acute cardiac[28] or kidney[29] injuries and hence, increase the risk of a fatal outcome associated with COVID-19[30]. People with obesity have been previously characterized by systemic low-grade inflammation, impaired immune response to infections, and higher susceptibility and mortality associated with infections[17,31,32]. These factors may all lead to a greater mortality risk in those who contracted SARS-CoV-2. Several studies emerging from different countries identified obesity as an independent risk factor for hospitalisation and death due to COVID-19[11,12,18,31,32], with a BMI ≥ 35 kg/m2 radically increasing the mortality risk[33].

In our study, asthma diagnosis was present among 7.6% of hospitalized patients with COVID-19, which is lower than the 9.8% prevalence of asthma in Northern Ireland[34]. At the same time, we found asthma to be associated with a lower risk of COVID-19-related death in the full sample, although the results of our sensitivity analysis showed no difference in risk of COVID-19-related death in patients with asthma compared to individuals with no documented comorbidities. This finding supports the mixed evidence on the role of asthma in influencing COVID-19-related outcomes. The OpenSAFELY study identified asthma as a significant risk factor of death in patients with COVID-19 and indicated that patients on inhaled corticosteroids have the greatest risk[3]. In vitro studies have suggested that corticosteroids use can result in

impaired antiviral innate immune responses[35,36] and delayed virus clearance[37], and this in turn can potentially lead to more severe outcomes in individuals who contracted SARS-CoV-2; however, this hypothesis has to be further tested. Several studies found no statistically significant difference in mortality risk by asthma status[38,39]. For example, the prospective case-control study based on the UK Biobank data showed that asthma did not significantly increase the odds of COVID-19 mortality[38]. More recent work however indicated that people with asthma were in fact less likely to die due to COVID-19[40]. Interestingly, the risk of severe clinical outcomes of COVID-19 was lower in people with allergic asthma[41]. Akenroye et al.[42] suggested that some asthma medications, such as mepolizumab, reslizumab, and benralizumab, may enhance immune responses to viral infections and potentially decrease susceptibility to additional lung injury from diseases such as COVID-19. The association between asthma and COVID-19 mortality could also differ by the degree of asthma severity[3]. Given that our sensitivity analysis showed no difference in risk of COVID-19-related death in patients with asthma compared to individuals with no documented comorbidities, but our primary results indicated a lower risk of COVID-19-related death in patients with asthma compared to non-asthma controls, further analysis is required to evaluate the impact of differing patterns and extents of multimorbidity in patients with asthma (e.g. cardiometabolic multimorbidity) and the role of different asthma medications when examining COVID-19-related outcomes.

We confirmed that COVID-19-related mortality increased with older age. In particular, patients in age groups ≥ 50 years old had higher odds of COVID-19-related death when compared with those aged 40 to 49 years. Higher COVID-19 mortality among older adults has been known since early in the pandemic and has been described in detail[3,27,43]. The analysis based on the US epidemiologic data demonstrated that the overall COVID-19 case-fatality rate among individuals infected with SARS-CoV-19 was highest in those aged ≥85 years (range 10%–27%), followed by those aged 65-84 years (3%–11%), aged 55-64 years (1%–3%), and aged

<55 years (<1%)[43]. Greater risk of death due to COVID-19 in older adults is likely related to their declining immune defences, however other hypotheses have also been suggested[44].

Finally, we demonstrated that multimorbidity is an important clinical characteristic to consider in the context of the COVID19 pandemic. Our findings showing the higher COVID-19 in-hospital mortality risk in people with multiple underlying conditions are consistent with other published data[45]. A cross-sectional, multicenter, observational study of Italian COVID-19 population found that increasing multimorbidity, measured by the Charlson Comorbidity Index, was strongly associated with COVID-19-related death[46]. Kim et al.[47], in their study of 2,491 COVID-19 patients, reported that individuals with 3 or more underlying conditions had a 1.8 times higher risk of in-hospital mortality than patients with no underlying conditions. Multimorbidity was also reported to be a predictor of the risk of COVID-19 infection in a large UK Biobank cohort of 428,199 participants; however, the authors did not report on the relationship between the co-existence of multiple underlying conditions and risk of death[48].

We also analysed multimorbidity patterns by identifying clusters of conditions in hospitalised COVID-19 patients using an unsupervised machine learning technique (k-mode clustering). Our study revealed recognisable co-occurrences of COVID-19 with different combinations of diseases, with a potentially causal link or underlying mechanism, including cardiovascular diseases, respiratory diseases, mental and neurological disorders, metabolic and endocrine diseases, and renal diseases. For example, Cluster 1 characterized by the group of individuals with cardiovascular diseases had also a high percentage of cases with COPD. Previous studies suggested that the systemic inflammatory response associated with COPD may act as a possible mechanism that links COPD with increased risk for cardiovascular diseases[49]. Furthermore, it was demonstrated that COPD is associated with increased carotid intimal medial thickness (CIMT) and that among those with COPD, CIMT is linked to higher cardiovascular mortality[50]. Evidence also supports the association between renal disease and cognitive impairment (Cluster 2); however, the mechanisms underlying this association are not completely elucidated[51]. Although, direct impact of uremic toxins has been proposed as a

potential cause of cognitive decline, studies showed that dialysis prescription did not reverse symptoms of cognitive impairment[52]. Co-occurrence of other conditions, such as, i) metabolic syndrome (defined by the presence of metabolic abnormalities including obesity, glucose intolerance, and elevated blood pressure), cardiovascular disease, and liver disease (Cluster 2, 3 and 5)[53,54]; ii) mental disorders and heart disease (Cluster 6)[55], and iii) cancer with obesity and diabetes (Cluster 4)[56] has also been broadly documented and shown to be associated with high mortality. Therefore, the identification of these multimorbidity patterns among hospitalized individuals with COVID-19 can help identify opportunities to target patient-centred care towards people with high-risk ages and a specific combination of health conditions, leading to improved clinical outcomes. Note that we acknowledge that a presence of a specific chronic disease or a combination of chronic diseases in our analytic sample may have acted as an effect modifier of COVID-19 death but could also be associated with COVID-19 death via the existence of another common cause i.e., other clinical characteristics or socio-economic factors not included in our study.

The strengths of our study include the large, regional cohort of hospitalised COVID-19 patients, providing high statistical power to investigate associations between different risk factors and COVID-19 in-hospital mortality. The use of ICD-10 diagnosis codes assigned by medical professionals working in all hospitals throughout Northern Ireland meant that comprehensive information on a wide range of comorbidities were available. Our results remained robust in a number of sensitivity analyses and reinforced previous findings of a higher risk of COVID-19-related death associated with obesity[12], liver disease[57], renal disease[5], metastatic cancer[58], congestive heart failure[59], and COPD[19]. Furthermore, we investigated the associations between several conditions for which little data exist regarding risk for in-hospital mortality in patients with COVID-19, such as hypothyroidism, solid tumour without metastasis, lymphoma, dementia, and other neurological disorders. We also added to the, so far inconclusive, evidence on the role of asthma in influencing COVID-19-related outcomes. Finally, given a paucity of research on the impact of multimorbidity on the risk of

COVID-19 in-hospital mortality, we examined the association between increasing multimorbidity, multimorbidity patterns, and COVID-19-related death. To our knowledge, this is the first study to characterise patterns of multimorbidity in a hospitalised population with COVID 19 using an unsupervised machine learning approach.

The interpretation of our results should be made considering several limitations. First, due to unavailability of ICD-10 diagnosis codes, we were unable to consider the records of 552 patients with COVID-19, admitted to hospital in the period from March 1, 2020, to January 31, 2021. Furthermore, we have only used the comorbidity data collected in the studied period. Therefore, it is possible we missed some comorbidities by not including information reported at prior admissions. Second, the underlying cause of death was allocated based on the hospital records of ICD-10 diagnostic coding and discharge status, not death certificate; this might have led not only to the underestimation of the real magnitude of mortality due to COVID-19 but also potential misclassification of deaths from other causes. To assess the extent to which these inaccuracies may have affected our estimates, a similar analysis should be performed in the future using death certificate–based ICD-10 diagnosis codes. Third, since the ICD-10 diagnosis codes were assigned by medical professionals working in different hospitals, they may not have captured intended disease concepts with complete consistency. Fourth, our analysis is cross-sectional, meaning that both the independent variables and the outcome were collected simultaneously. Although the central element of the cross-sectional design is the lack of the temporal information required to describe the evolution of the underlying dynamics, in case of our study, the temporal link between the outcome and independent variables can be cautiously assumed since the presence of underlying health conditions most likely preceded the outcome studied (i.e., fatal/non-fatal hospitalization due to COVID-19). Fifth, the impact of inpatient treatment or procedures performed during hospitalisation as well as the information on the duration of the long-term conditions on patient outcomes was not considered in this study. In addition, unavailability of data on clinical parameters such as oxygen and ventilation treatment limited the opportunity of a more

comprehensive analysis including multiple levels of severity as the outcome. Sixth, the purpose of using the clustering approach was for this method to potentially become part of the pipeline for discovering the various multimorbidity profiles that are associated with mortality due to COVID-19. Extensions to this research may include a more robust look at the effects of the hyperparameters and different analytic samples on the associations between different disease groups and COVID-19-related deaths. Furthermore, the results of the clustering algorithm should be further validated by domain experts to determine its clinical utility. It is important to highlight that this analysis was performed among a cohort of individuals hospitalized with COVID-19 and may not be generalizable to those non-hospitalized. The lack of data on non-hospitalized individuals with SARS-CoV-2 infection limited our ability to examine utilization of our algorithm outside of the hospitalized patients. However, our population represented a diverse cohort with a variety of comorbidities and is representative of high-risk individuals across Northern Ireland. Seventh, as mentioned above, our analytic sample does not represent a true random selection from the population since it is solely based on hospitalised patients, that died or did not die during the admission episode following COVID-19 diagnosis. This non-randomness of sample selection may have had an impact on our study results. Older adults, those obese, and with pre-existing medical conditions are more susceptible to adverse COVID-19 outcomes, while COVID-19 severity likely influences hospitalisation[60]. As such, characteristics related to our sample inclusion, also relate to the hypothesised risk factors and the outcome of interest and hence, investigating these factors within hospitalised patients may have introduced the possibility of collider bias. In future work, the likelihood and extent of collider bias associated with sample selection could be evaluated by comparing means, variances, and distributions of variables in the sample of individuals with COVID-19 that have been hospitalized with those in a representative sample of the NI population[61]. This could not be evaluated in the current data set. Finally, our study may be subject to other potential sources of bias. For example, selection bias could have been introduced by not including non-hospitalized individuals living in long-term care or assisted living facilities that suffered from severe COVID-19 symptoms and subsequently died due to

COVID-19[62]. Bias due to omission of a confounder from the model (unmeasured confounder) is also a possibility. Several demographic factors (e.g., race/ethnicity, disability status, socio-economic status), as well as structural factors (e.g., literacy) were not included in the analysis[63-66]. Moreover, we did not control for the effect of concurrently prescribed medications such as antipsychotics, proton pump inhibitors, antihistamines, and opioid analgesics that were previously shown to increase the risk of adverse outcomes among patients with COVID-19[67]. The association between certain drug classes, polypharmacy, and the risk of COVID-19 mortality should be addressed in future work. In addition, further analysis on host-specific genetic factors and their relationships with severe manifestations of COVID-19, in particular, in individuals with no underlying health conditions that died as a direct consequence of SARS-CoV-2 infection, could allow us to better understand relationships between environmental risk factors and severe outcomes associated with COVID-19 and identify potential targets for therapeutic development.

This study estimated the impact of the broad spectrum of comorbidities on COVID-19 in-hospital mortality using a large, regional cohort of hospitalized patients. We found that individuals with lymphoma, metastatic cancer, solid tumour without metastasis, liver disease, congestive heart failure, chronic obstructive pulmonary disease, obesity, renal disease, and dementia were at significantly increased risk for COVID-19-related death. In addition, we showed that the presence of multiple coexisting health conditions further increased odds of death. Given that effective clinical management of patients with multimorbidity is therefore a critical step towards their survival from COVID-19, multidisciplinary clinical teams should prepare comprehensive care plans that can be streamlined and meet the dynamic needs of such patients. Future work should investigate the associations between different patterns of multimorbidity and COVID-19 mortality to better characterize those individuals who would benefit from enhanced preventive measures.

Methods

Study design and study population

This retrospective cohort study was conducted using the Patient Administration System (PAS) data from Northern Ireland, covering the period from March 1, 2020, to January 31, 2021. The PAS data includes patient level data on hospital admissions, transfers, and discharges for all NHS Trusts in Northern Ireland. In total, 8524 records identified as COVID-19 hospital admissions (using PAS admission codes) were initially selected for analysis (Fig.1). We then excluded n = 1075 patients that were not clinically, epidemiologically, or laboratory-confirmed COVID-19 cases as indicated by COVID-19-related ICD-10 diagnostic codes of U07.1 and U07.2, leaving 7449 patients eligible for analysis. Next, we excluded n = 552 patients for whom ICD-10 codes of U07.1 and U07.2 were not recorded. In effect, analyses were restricted to n = 6897 patients with ICD-10 codes for COVID-19, including the primary or secondary diagnosis code of U07.1 and U07.2, with complete ICD-10 data on underlying conditions. Finally, we combined the ICD-10 diagnosis codes from multiple hospitalizations due to COVID-19, including transfers between or within hospitals, and removed the duplicate ICD-10 codes (n = 861). Note that the discharge code of the most recent hospital stay was used for patients with multiple records of hospital admissions. This resulted in a subset of 6036 hospitalized COVID-19 cases included in the analysis. Specifically, we compared characteristics of COVID-19 patients that were discharged from hospitals (n = 4811) with those that died in hospital during the admission episode following COVID-19 diagnosis (n = 1225). Fig. 1. shows the stages of the selection process.

Outcomes

The outcome used in our analysis were hospital discharge and in-hospital COVID-19-related death, defined on the basis of a discharge status and the International Classification of Diseases (version 10; ICD-10) code diagnosis, of U07.1 (“COVID-19, virus identified”) or U07.2 (“COVID-19, virus not identified”), recorded as either a primary or secondary diagnosis to ensure the inclusion of all people who had COVID-19 in hospital[68]. Specifically, U07.1 code was assigned when COVID-19 was confirmed by laboratory testing irrespective of severity of

clinical signs or symptoms while U07.2 code was used when COVID-19 was diagnosed clinically or epidemiologically but laboratory testing was inconclusive or not available. For patients with multiple records of hospital admissions identified as COVID-19 hospital admissions, including those transferred between or within hospitals, the discharge code of the most recent hospital stay, was considered the outcome measure. Therefore, the same person was not included more than once in the analytic sample while all in-hospital COVID-19-related deaths were counted. The presence of specific comorbidities in patients with multiple records was established by combining the ICD-10 diagnosis codes from all visits and removing the duplicate codes.

Covariates

We examined the following patient-level data for each admission: (i) demographics (age, sex); and (ii) ICD-10 diagnosis codes that were used to identify common comorbidities. The presence of specific comorbidities in patients with multiple records of hospital admissions, including those transferred between or within hospitals, was established by combining the ICD-10 diagnosis codes from all considered visits and removing the duplicate codes. Subsequently, the discharge code of the most recent hospital stay for a patient with multiple records of hospital admissions was considered the outcome measure. The ICD-10 coding criteria for all comorbidities were adapted from the Elixhauser comorbidity measure[69]. Accordingly, the following comorbidities were considered: congestive heart failure (I09.9, I11.0, I13.0, I13.2, I25.5, I42.0, I42.5 - I42.9, I43.x, I50.x, P29.0), cardiac arrhythmias (I44.1 - I44.3, I45.6, I45.9, I47.x - I49.x, R00.0, R00.1, R00.8, T82.1, Z45.0, Z95.0), chronic obstructive pulmonary disease (I27.8, I27.9, J40.x - J47.x, J60.x - J67.x, J68.4, J70.1, J70.3), diabetes including uncomplicated (E10.0, E10.1, E10.9, E11.0, E11.1, E11.9, E12.0, E12.1, E12.9, E13.0, E13.1, E13.9, E14.0, E14.1, E14.9) and complicated cases (E10.2 - E10.8, E11.2 - E11.8, E12.2 - E12.8, E13.2 - E13.8, E14.2 - E14.8), hypertension (I10.x, I11.x - I13.x, I15.x), hypothyroidism (E00.x - E03.x, E89.0), renal disease (I12.0, I13.1, N03.2 - N03.7, N05.2 - N05.7, N18.x, N19.x, N25.0, Z49.0 - Z49.2, Z94.0, Z99.2), liver disease (B18.x, I85.x, I86.4,

I98.2, K70.x, K71.1, K71.3 - K71.5, K71.7, K72.x - K74.x, K76.0, K76.2 - K76.9, Z94.4), lymphoma (C81.x - C85.x, C88.x, C96.x, C90.0, C90.2), metastatic cancer (C77.x - C80.x), solid tumour without metastasis (C00.x - C26.x, C30.x - C34.x, C37.x - C41.x, C43.x, C45.x - C58.x, C60.x - C76.x, C97.x), rheumatoid arthritis/collagen vascular diseases (L94.0, L94.1, L94.3, M05.x, M06.x, M08.x, M12.0, M12.3, M30.x, M31.0 - M31.3, M32.x - M35.x, M45.x, M46.1, M46.8, M46.9), obesity (E66.x), depression (F20.4, F31.3 - F31.5, F32.x, F33.x, F34.1, F41.2, F43.2), dementia (F00.x - F03.x, F05.1, G30.x, G31.1), neurological disorders such as paralysis (G04.1, G11.4, G80.1, G80.2, G81.x, G82.x, G83.0 - G83.4, G83.9) and other neurological disorders (G10.x - G13.x, G20.x - G22.x, G25.4, G25.5, G31.2, G31.8, G31.9, G32.x, G35.x - G37.x, G40.x, G41.x, G93.1, G93.4, R47.0, R56.x), and asthma (J.45).

Statistical analysis

Descriptive statistics were provided using median (interquartile range [IQR]) for continuous variables and counts and percentages for categorical data. The Shapiro-Wilk test was used to determine if the data deviates from a normal distribution. The Wilcoxon rank sum test was applied to compare groups of continuous data. Statistical differences between groups of categorical variables were established with the Fisher test. A two-sided $P < 0.05$ was considered statistically significant.

The association between patient characteristics and in-hospital mortality was examined using logistic regression models. Adjusted logistic regression analyses were presented as odds ratios (OR) with 95% confidence intervals (95%CI) and included age, sex, and pre-existing long-term conditions identified using ICD-10 coding rules. Statistical significance was determined using the Wald's test. Non-linear dependence between age and the outcome measure was evaluated by comparing the generalised additive model with linear and spline terms for age against the model with only a linear term. The likelihood-ratio $\chi 2$ test indicated that linearity assumption was not satisfied ($p < 0.001$). We therefore grouped age into six categories (0-40, 40-50, 50-60, 60-70, 70-80 and ≥80 years) since odds ratios calculated for variables modelled as polynomials in the model, are not directly interpretable. Additionally, we provided the odds ratios adjusted for age parametrised as a 4-knot restricted cubic spline in the Supplementary Material (Table S1 in Supplementary Material). Furthermore, the non-linear relationship between age and the risk of death involving COVID-19 is shown in Fig. F1 in Supplementary Material.

We investigated the potential effect of having multiple pre-existing long-term conditions on the outcome measure (hospital discharge vs. in-hospital COVID-19-related death). The analysis was conducted for the total population and stratified by sex. Comorbidities were expressed as categorical variables from 0 to 2, then 3 or higher. The model including categories of comorbidities was adjusted for age and sex in the analysis including all patients and age only

in the sex-stratified analysis, with age modelled as a polynomial in the logistic regression model.

We further investigated patterns of multimorbidity by performing a cluster analysis. First, we classified all considered health conditions into eight groups: 1) autoimmune diseases including rheumatoid arthritis and collagen vascular diseases; 2) metabolic and endocrine diseases including diabetes, hypothyroidism, and obesity; 3) respiratory diseases including chronic obstructive pulmonary disease and asthma; 4) cardiovascular diseases including hypertension, congestive heart failure, and cardiac arrhythmias; 5) mental and neurological disorders including depression, dementia, and neurological disorders; 6) neoplasms including lymphoma, metastatic cancer, and solid tumour without metastasis; 7) digestive system diseases including liver disease; and 8) genitourinary system diseases including renal diseases. To identify disease clusters within the analytic sample, we used the k-modes clustering approach, an unsupervised machine learning algorithm for grouping categorical data[70]. The method partitions the objects (patients) into a specified number of clusters such that the distance from objects to the assigned cluster modes is minimized. We used the Hamming distance to determine the dissimilarity of two objects. The optimal number of clusters was determined with the modified Elbow method using within-cluster differences[71]. Frequencies and percentages of disease groups were then calculated for each cluster. Descriptive statistics of age and in-hospital mortality in patients with COVID-19 were also provided for each cluster. All analyses were conducted using R Studio v.1.2.5033 operating R v.3.6.3.

Sensitivity analysis

Four sensitivity analyses were performed to validate the robustness of the main findings. First, COVID-19-related deaths were defined as COVID-19 related admissions with ICD-10 code diagnosis, including only primary or secondary diagnosis code of U07.1 which is likely to have a greater degree of specificity. Second, COVID-19-related deaths were defined as COVID-19 related admissions with ICD-10 diagnosis code U07.1 or U07.2 as the primary code

for clinical diagnosis. Note that U07.1 code represents cases when COVID-19 was confirmed by laboratory testing while U07.2 code corresponds to COVID-19 cases that were diagnosed clinically or epidemiologically since laboratory testing was inconclusive or not available. Third, to assess the relative importance of underlying health conditions to adverse outcomes, we compared the risk of death from COVID-19 in individuals with comorbidity and those with no recorded comorbidity, adjusting for sex and age parametrised as a 4-knot restricted cubic spline. Finally, since asthma was found to be associated with a decreased risk of COVID-19-related death, we also performed a fourth sensitivity analysis to compare the risk of death from COVID-19 in people with asthma and those with no documented comorbidities. The model was adjusted for sex and age parametrised as a 4-knot restricted cubic spline.

Ethical considerations

The study and data collection were approved by the Office for Research Ethics Committees Northern Ireland (20/0062). We followed the ethical standards set by the Helsinki Declaration of 1964, as revised in 2013, and the research guidelines of the University of Ulster. The Office for Research Ethics Committees Northern Ireland considered the collection of routine data as evaluation of service and waived the need for written informed consent. The Office for Research Ethics Committees Northern Ireland approved the publication of results. We followed the Strengthening the Reporting of Observational Studies in Epidemiology (STROBE) guidelines[72].

References

1. Guan, W.J. et al. Clinical characteristics of coronavirus disease 2019 in China. *New England journal of medicine*. 382(18), 1708-1720 (2020).
2. Sun, P. et al. Clinical characteristics of hospitalized patients with SARS-CoV-2 infection: a single arm meta-analysis. *Journal of medical virology*. 92(6), 612-617 (2020).
3. Williamson, E.J. et al. Factors associated with COVID-19-related death using OpenSAFELY. *Nature*. 584(7821), 430-436 (2020).
4. Deng, G., Yin, M., Chen, X., Zeng, F. Clinical determinants for fatality of 44,672 patients with COVID-19. *Critical Care*. 24, 1-3 (2020).
5. Docherty, A.B. et al. Features of 20 133 UK patients in hospital with covid-19 using the ISARIC WHO Clinical Characterisation Protocol: prospective observational cohort study. *BMJ*. 369 (2020).
6. Bae, S. et al. Impact of cardiovascular disease and risk factors on fatal outcomes in patients with COVID-19 according to age: a systematic review and meta-analysis. *Heart*. 107(5), 373-380 (2021).
7. Chen, T. et al. Clinical characteristics of 113 deceased patients with coronavirus disease 2019: retrospective study. *BMJ*. 368 (2020).
8. Yang, J. et al. Prevalence of comorbidities in the novel Wuhan coronavirus (COVID-19) infection: a systematic review and meta-analysis. *Int J Infect Dis*.10 (2020).
9. Meng, Y. et al. Cancer history is an independent risk factor for mortality in hospitalized COVID-19 patients: a propensity score-matched analysis. *Journal of Hematology & Oncology*. 2020;13(1):1-1.
10. Henry, B.M., Lippi, G. Chronic kidney disease is associated with severe coronavirus disease 2019 (COVID-19) infection. *International Urology and Nephrology*. 52(6), 1193-1194 (2020).
11. Lighter, J. et al. Obesity in patients younger than 60 years is a risk factor for Covid-19 hospital admission. *Clinical Infectious Diseases*. 71(15), 896-897 (2020).

12 Dietz, W., Santos-Burgoa, C. Obesity and its implications for COVID-19 mortality. *Obesity*. 28(6):1005 (2020).

13 National Records of Scotland. *Deaths involving coronavirus (COVID-19) in Scotland*. (2021).

14 Chen, N. et al. Epidemiological and clinical characteristics of 99 cases of 2019 novel coronavirus pneumonia in Wuhan, China: a descriptive study. *The Lancet*. 395(10223), 507-513 (2020).

15 Wu, Z., McGoogan, J.M. Characteristics of and important lessons from the coronavirus disease 2019 (COVID-19) outbreak in China: summary of a report of 72 314 cases from the Chinese Center for Disease Control and Prevention. *JAMA*. 323(13), 1239-1242 (2020).

16 Docherty, A. B. et al. Features of 20 133 UK patients in hospital with covid-19 using the ISARIC WHO Clinical Characterisation Protocol: prospective observational cohort study. *BMJ*, 369, m1985, 10.1136/bmj.m1985 (2020).

17 Popkin, B.M. et al. Individuals with obesity and COVID-19: A global perspective on the epidemiology and biological relationships. *Obesity Reviews*. 21(11), e13128 (2020).

18 Simonnet, A. et al. LICORN and the Lille COVID-19 and Obesity Study Group. High prevalence of obesity in severe acute respiratory syndrome coronavirus-2 (SARS-CoV-2) requiring invasive mechanical ventilation. Obesity. 28(7), 1195-1199 (2020).

19 Gerayeli, F.V. et al. COPD and the risk of poor outcomes in COVID-19: A systematic review and meta-analysis. *EClinicalMedicine*. 33, 100789 (2021).

20 Alqahtani J.S. et al. Prevalence, severity and mortality associated with COPD and smoking in patients with COVID-19: a rapid systematic review and meta-analysis. *PloS One*. 15(5), e0233147 (2020).

21 Rabbani, G. et al. Pre-existing COPD is associated with an increased risk of mortality and severity in COVID-19: a rapid systematic review and meta-analysis. *Expert Review of Respiratory Medicine*.15(5), 705-716 (2021).

22 Wang, Q., Davis, P.B., Gurney, M.E., Xu, R. COVID-19 and dementia: Analyses of risk, disparity, and outcomes from electronic health records in the US. *Alzheimer's & Dementia*. 7(8), 1297-1306 (2021).

23 Tahira, A.C., Verjovski-Almeida, S., Ferreira, S.T. Dementia is an age-independent risk factor for severity and death in COVID-19 inpatients. *Alzheimer's & Dementia*. 17(11), 1818-1831 (2021).

24 Kuo, C.L. et al. APOE e4 genotype predicts severe COVID-19 in the UK Biobank community cohort. *The Journals of Gerontology: Series A*. 75(11), 2231-2232 (2020).

25 Sweeney, M.D., Sagare, A.P., Zlokovic, B.V. Blood–brain barrier breakdown in Alzheimer disease and other neurodegenerative disorders. *Nat Rev Neurol*. 14(3), 133 (2018).

26 Ueno, M. et al. Blood-brain barrier damage in vascular dementia. *Neuropathology*. 36(2), 115-124 (2016).

27 Reilev, M,. et al. Characteristics and predictors of hospitalization and death in the first 11 122 cases with a positive RT-PCR test for SARS-CoV-2 in Denmark: a nationwide cohort. *International Journal of Epidemiology*. 49(5), 1468-1481 (2020).

28 Tomasoni, D. et al. COVID-19 and heart failure: from infection to inflammation and angiotensin II stimulation. Searching for evidence from a new disease. *European Journal of Heart Failure*. 22(6), 957-66 (2020).

29 Hirsch, J.S. et al. Acute kidney injury in patients hospitalized with COVID-19. *Kidney International*. 98(1), 209-218 (2020).

30 Yang, R., Gui, X., Zhang, Y., Xiong, Y. The role of essential organ-based comorbidities in the prognosis of COVID-19 infection patients. *Expert Review of Respiratory Medicine*. 14(8), 835-838 (2020).

31 Sattar, N., McInnes, I.B., McMurray, J.J.V. Obesity a risk factor for severe COVID-19 infection: multiple potential mechanisms. *Circulation*. 142(1), 4–6 (2020).

32 Caussy, C., Wallet, F., Laville, M., Disse, E. Obesity is associated with severe forms of COVID-19. *Obesity.* 28(7), 1175 (2020).

33 Rottoli, M. et al. How important is obesity as a risk factor for respiratory failure, intensive care admission and death in hospitalised COVID-19 patients? Results from a single Italian Centre. *Eur J Endocrinol*. 183(4), 389–397 (2020).

34 Asthma UK. *Asthma facts and statistics*. https://www.asthma.org.uk/about/media/facts-and-statistics/ (2017).

35 Davies, J.M. et al. Budesonide and formoterol reduce early innate anti-viral immune responses in vitro. *PLoS One*. 6(11), e27898 (2011).

36 Simpson J.L. et al. Reduced antiviral interferon production in poorly controlled asthma is associated with neutrophilic inflammation and high-dose inhaled corticosteroids. *Chest*. 149(3), 704-713 (2016).

37 Singanayagam, A. et al. Corticosteroid suppression of antiviral immunity increases bacterial loads and mucus production in COPD exacerbations. *Nature Communications*. 9(1), 1-6 (2018).

38 Khawaja, A.P. et al. Associations with covid-19 hospitalisation amongst 406,793 adults: the UK Biobank prospective cohort study. MedRxiv. 10.1101/2020.05.06.20092957 (2020).

39 Sunjaya, A.P., Allida, S.M., Di Tanna, G.L., Jenkins, C.R. Asthma and COVID-19 risk: a systematic review and meta-analysis. *European Respiratory Journal*, 59(3) (2022).

40 Hou, H., Xu, J., Li, Y., Wang, Y., Yang, H. The association of asthma with COVID-19 mortality: an updated meta-analysis based on adjusted effect estimates. *The Journal of Allergy and Clinical Immunology: In Practice*, 9(11), 3944-3968 (2021).

41 Zhu, Z. et al. Association of asthma and its genetic predisposition with the risk of severe COVID-19. *Journal of Allergy and Clinical Immunology.* 146(2), 327-329 (2020).

42 Akenroye, A.T., Wood, R., Keet, C. Asthma, biologics, corticosteroids, and coronavirus disease 2019. *Ann Allergy Asthma Immunol.* 125(1), 12-13 (2020).

43 Petersen, E. et al. Comparing SARS-CoV-2 with SARS-CoV and influenza pandemics. *The Lancet Infectious Diseases*, 20(9), e238-e244 (2020).

44 Mueller, A.L., McNamara, M.S., Sinclair, D.A. Why does COVID-19 disproportionately affect older people? *Aging.* 12(10), 9959 (2020).

45 Fernández-Niño, J.A., Guerra-Gómez, J.A. and Idrovo, A.J., 2020. Multimorbidity patterns among COVID-19 deaths: proposal for the construction of etiological models. *Rev Panam Salud Publica*. 44:e166 (2020).

46 Iaccarino, G. et al. Age and multimorbidity predict death among COVID-19 patients: results of the SARS-RAS study of the Italian Society of Hypertension. *Hypertension.* 76(2), 366-372 (2020).

47 Kim, L. et al. Risk factors for intensive care unit admission and in-hospital mortality among hospitalized adults identified through the US coronavirus disease 2019 (COVID-19)-associated hospitalization surveillance network (COVID-NET). *Clinical Infectious Diseases.* 72(9), e206-e214 (2021).

48 McQueenie, R. et al. Multimorbidity, polypharmacy, and COVID-19 infection within the UK Biobank cohort. *PloS One*. 15(8), e0238091 (2020).

49 Sin, D.D., Man, S.P. Why are patients with chronic obstructive pulmonary disease at increased risk of cardiovascular diseases? The potential role of systemic inflammation in chronic obstructive pulmonary disease. *Circulation*. 107(11), 1514-1519 (2003).

50 Maclay, J.D., MacNee, W. Cardiovascular disease in COPD: mechanisms. *Chest*. 143(3), 798-807 (2013).

51 Silva, S.T.D., Ribeiro, R.D.C.L., Rosa, C.D.O.B., Cotta, R.M.M. Cognitive capacity in individuals with chronic kidney disease: relation to demographic and clinical characteristics. *Brazilian Journal of Nephrology*. 36, 163-170 (2014).

52 Radic, J. et al. The possible impact of dialysis modality on cognitive function in chronic dialysis patients. *Neth J Med.* 68(4), 153-7 (2010).

53 Cheong, K.C. et al. Association of metabolic syndrome with risk of cardiovascular disease mortality and all-cause mortality among Malaysian adults: a retrospective cohort study. *BMJ Open*. 11(8), e047849 (2021).

54 Käräjämäki, A.J. et al. Non-alcoholic fatty liver disease with and without metabolic syndrome: different long-term outcomes. *Metabolism.* 66, 55-63 (2017).

55 De Hert, M., Detraux, J., Vancampfort, D. The intriguing relationship between coronary heart disease and mental disorders. Dialogues in clinical neuroscience. 20(1), 31–40 (2022).

56 Hawkins, M.L. wt al. Endocrine and metabolic diseases among colorectal cancer survivors in a population-based cohort. *JNCI: Journal of the National Cancer Institute.* 112(1), 78-86 (2020).

57 Galiero, R. et al. Impact of chronic liver disease upon admission on COVID-19 in-hospital mortality: Findings from COVOCA study. *PloS One.* 15(12), e0243700 (2020).

58 Lee, L.Y. et al. COVID-19 prevalence and mortality in patients with cancer and the effect of primary tumour subtype and patient demographics: a prospective cohort study. *The Lancet Oncology.* 21(10), 1309-1316 (2020).

59 Doolub, G. et al. Impact of COVID-19 on inpatient referral of acute heart failure: a single-centre experience from the south-west of the UK. *ESC Heart Failure.* 8(2), 1691-1695 (2021).

60 de Lusignan, S. et al. Risk factors for SARS-CoV-2 among patients in the Oxford Royal College of General Practitioners Research and Surveillance Centre primary care network: a cross-sectional study. *The Lancet Infectious Diseases*, *20*(9), 1034-1042 (2020).

61 Griffith, G.J. et al. 2020. Collider bias undermines our understanding of COVID-19 disease risk and severity. *Nature communications*, 11(1), 1-12 (2020).

62 The Northern Ireland Statistics and Research Agency. *Covid-19 related deaths and pre-existing conditions in Northern Ireland: March 2020 to November 2021.* nisra.gov.uk/sites/nisra.gov.uk/files/publications/C19-PEC-Nov21_0.pdf (2021).

63 Raleigh, V.S. Ethnic differences in covid-19 death rates. *BMJ*. 376 (2022).

64 Bosworth, M.L. et al. Deaths involving COVID-19 by self-reported disability status during the first two waves of the COVID-19 pandemic in England: a retrospective, population-based cohort study. *The Lancet Public Health*. *6*(11), e817-e825 (2021).

65 Cifuentes, M.P., Rodriguez-Villamizar, L.A., Rojas-Botero, M.L., Alvarez-Moreno, C.A. and Fernández-Niño, J.A. Socioeconomic inequalities associated with mortality for COVID-19 in Colombia: a cohort nationwide study. *J Epidemiol Community Health*. *75*(7), 610-615 (2021).

66 Paakkari, L. and Okan, O. COVID-19: health literacy is an underestimated problem. *The Lancet Public Health*. *5*(5), e249-e250 (2020).

67 McKeigue, P.M. et al. Relation of severe COVID-19 to polypharmacy and prescribing of psychotropic drugs: the REACT-SCOT case-control study. *BMC Medicine*. *19*(1), 1-11 (2021).

68 Quan, H. et al. Coding algorithms for defining comorbidities in ICD-9-CM and ICD-10 administrative data. *Medical Care.* 43(11), 1130-1139 (2005).

69 van Walraven, C., Austin, P.C., Jennings, A., Quan, H., Forster, A.J. A modification of the Elixhauser comorbidity measures into a point system for hospital death using administrative data. *Medical Care.* 47(6), 626-633 (2009).

70 Huang, Z. A fast clustering algorithm to cluster very large categorical data sets in data mining. *In KDD: Techniques and Applications* (H. Lu, H. Motoda and H. Luu, Eds.). 21-34, World Scientific, Singapore (1997).

71 Shalev-Shwartz, S. and Ben-David, S. Understanding machine learning: From theory to algorithms. Cambridge University Press (2014).

72 Vandenbroucke, J.P. et al. Strobe Initiative. Strengthening the Reporting of Observational Studies in Epidemiology (STROBE): explanation and elaboration. *PLoS Medicine*. 4(10), e297 (2007).

Acknowledgements

The authors wish to thank the Information Team, Performance Management Service Improvement Directorate, HSCB for assisting in the collation of the data.

Author contributions

MB, AJB, HVW conceptualized the study. MB analysed the data. MB, DeB, DaB, LP, DG, AJB contributed to interpretation of the results. MB took the lead in writing the first draft. MB, DeB, DaB, LP, RS, HVW, DG, AJB critically reviewed the manuscript. All authors approved the final manuscript.

Competing interests

The authors declare no competing interests.

Data availability

De-identified study data are available for access by accredited researchers in accordance with data sharing policies of HSCB Performance Management Service Improvement Directorate.

Ethical considerations

The study and data collection were approved by the Office for Research Ethics Committees Northern Ireland (20/0062). We followed the ethical standards set by the Helsinki Declaration of 1964, as revised in 2013, and the research guidelines of the University of Ulster. The Office for Research Ethics Committees Northern Ireland considered the collection of routine data as evaluation of service and waived the need for written informed consent. The Office for Research Ethics Committees Northern Ireland approved the publication of results.

Figures

Fig. 1. Flow diagram indicating the selection of study participants.

Tables

Table 1. Demographic and clinical characteristics of patients.

	Discharged (n = 4811)	Died (n = 1225)	*P* value
Age, median (IQR)	68 (54-80)	80 (73-86)	**< 0.001**
Sex, n (%)			
Female	2309 (48.0)	514 (42.0)	**< 0.001**
Male	2502 (52.0)	711 (58.0)	
Congestive heart failure, n (%)			
No	4599 (95.6)	1095 (89.4)	**< 0.001**
Yes	212 (4.4)	130 (10.6)	
Cardiac arrhythmias, n (%)			
No	4253 (88.4)	1030 (84.0)	**< 0.001**
Yes	558 (11.6)	195 (16.0)	
Chronic obstructive pulmonary disease, n (%)			
No	3885 (80.8)	958 (78.2)	**0.049**
Yes	926 (19.2)	267 (21.8)	
Diabetes, n (%)			
No	4086 (84.9)	1007 (82.2)	**0.02**
Yes	725 (15.1)	218 (17.8)	
Hypertension, n (%)			
No	3738 (77.7)	927 (75.7)	0.14
Yes	1073 (22.3)	298 (24.3)	
Hypothyroidism, n (%)			
No	4629 (96.2)	1188 (97.0)	0.23
Yes	182 (3.8)	37 (3.0)	
Renal disease, n (%)			
No	4402 (91.5)	985 (80.4)	**< 0.001**
Yes	409 (8.5)	240 (19.6)	
Liver disease, n (%)			
No	4740 (98.5)	1198 (97.8)	0.08
Yes	71 (1.5)	27 (2.2)	
Lymphoma, n (%)			
No	4771 (99.2)	1198 (97.8)	**< 0.001**
Yes	40 (0.8)	27 (2.2)	
Metastatic cancer, n (%)			
No	4766 (99.1)	1192 (97.3)	**< 0.001**
Yes	45 (0.9)	33 (2.7)	
Solid tumour without metastasis, n (%)			
No	4700 (97.7)	1155 (94.3)	**< 0.001**
Yes	111 (2.3)	70 (5.7)	
Rheumatoid arthritis/collagen vascular diseases, n (%)			
No	4727 (98.3)	1203 (98.2)	0.9
Yes	84 (1.7)	22 (1.8)	
Obesity, n (%)			
No	4763 (99.0)	1204 (98.3)	**0.049**
Yes	48 (1.0)	21 (1.7)	
Asthma, n (%)			
No	4403 (91.5)	1173 (95.8)	**< 0.001**
Yes	408 (8.5)	52 (4.2)	
Depression, n (%)			
No	4583 (95.3)	1205 (98.4)	**< 0.001**
Yes	228 (4.7)	20 (1.6)	
Dementia, n (%)			
No	4467 (92.9)	1038 (84.7)	**< 0.001**
Yes	344 (7.1)	187 (15.3)	
Neurological disorders, n (%)			
No	4557 (94.7)	1153 (94.1)	
Yes	254 (5.3)	72 (5.9)	

Statistically significant $P < 0.05$ values are in bold.

Table 2. Multivariable logistic regression analysis for factors associated with COVID-19-related death.

Characteristic	Level	Number of deaths, n (%)	Adjusted odds ratio (95%CI)	*P* value
Age	40-49	10 (0.8)	reference	-
	0-39	5 (0.4)	0.60 (0.20,1.77)	0.36
	50-59	60 (4.9)	3.62 (1.83,7.17)	**< 0.001**
	60-69	176 (14.4)	8.98 (4.68,17.24)	**< 0.001**
	70-79	370 (30.2)	14.53 (7.62,27.69)	**< 0.001**
	≥ 80	604 (49.3)	23.77 (12.48,45.27)	**< 0.001**
Sex	Male	711 (58.0)	1.41 (1.22,1.62)	**< 0.001**
Congestive heart failure	Yes	130 (10.6)	1.69 (1.32,2.15)	**< 0.001**
Cardiac arrhythmias	Yes	195 (16.0)	0.92 (0.76,1.11)	0.38
COPD	Yes	267 (21.8)	1.43 (1.18,1.72)	**< 0.001**
Diabetes	Yes	218 (17.8)	1.13 (0.95,1.36)	0.17
Hypertension	Yes	298 (24.3)	0.94 (0.80,1.10)	0.43
Hypothyroidism	Yes	37 (3.0)	0.91 (0.62,1.34)	0.65
Renal disease	Yes	240 (19.6)	1.81 (1.51,2.19)	**< 0.001**
Liver disease	Yes	27 (2.2)	2.50 (1.53,4.07)	**< 0.001**
Lymphoma	Yes	27 (2.2)	2.78 (1.64,4.74)	**< 0.001**
Metastatic cancer	Yes	33 (2.7)	2.17 (1.25,3.77)	**0.006**
Solid tumour without metastasis	Yes	70 (5.7)	1.67 (1.16,2.41)	**0.006**
Rheumatoid arthritis/ collagen vascular diseases	Yes	22 (1.8)	1.08 (0.66,1.79)	0.78
Obesity	Yes	21 (1.7)	5.28 (2.92,9.52)	**< 0.001**
Asthma	Yes	52 (4.2)	0.60 (0.42,0.86)	**0.005**
Depression	Yes	20 (1.6)	0.62 (0.37,1.00)	0.05
Dementia	Yes	187 (15.3)	1.44 (1.17,1.76)	**< 0.001**
Neurological disorders	Yes	72 (5.9)	1.20 (0.90,1.61)	0.21

Statistically significant $P < 0.05$ values are in bold.
Observations, n = 6036, including 1225 COVID-19 deaths
CI: Confidence Interval; COPD: Chronic obstructive pulmonary disease

Table 3. Number of pre-existing health conditions and risk of COVID-19-related death

	ALL				MALES				FEMALES			
Pre-existing health conditions	Total number, n (%)	Number of deaths, n (%)	Adjusted odds ratio (95%CI[a])	*P* value	Total number, n (%)	Number of deaths, n (%)	Adjusted odds ratio (95%CI[b])	*P* value	Total number, n (%)	Number of deaths, n (%)	Adjusted odds ratio (95%CI[b])	*P* value
0	1713 (28.4)	187 (15.3)	reference		913 (28.4)	111 (15.6)	reference		800 (28.3)	76 (14.8)	reference	
1	2010 (33.3)	430 (35.1)	1.44 (1.18,1.75)	**< 0.001**	1104 (34.4)	245 (34.5)	1.34 (1.04,1.74)	**0.03**	906 (32.1)	185 (35.9)	1.56 (1.15,2.11)	**0.004**
2	1507 (25.0)	394 (32.2)	1.79 (1.47,2.20)	**< 0.001**	826 (25.7)	239 (33.6)	1.84 (1.41,2.40)	**< 0.001**	681 (24.1)	155 (30.2)	1.74 (1.27,2.38)	**< 0.001**
3 or more	806 (13.4)	214 (17.4)	1.80 (1.43,2.27)	**< 0.001**	370 (11.5)	116 (16.3)	2.00 (1.46,2.75)	**< 0.001**	436 (15.5)	98 (19.1)	1.63 (1.16,2.29)	**0.005**

[a] Adjusted odds ratios: adjusted for age and sex

[b] Adjusted odds ratios: adjusted for age

CI: Confidence Interval; COPD: Chronic obstructive pulmonary disease

Table 4. Multimorbidity clusters in a population of hospitalised patients with COVID-19. Values in bold correspond to three conditions with the highest prevalence within patient groups (clusters of conditions). Underlined values represent other, less common conditions that have a higher prevalence than in other groups.

	**Cluster 1**	**Cluster 2**	**Cluster 3**	**Cluster 4**	**Cluster 5**	**Cluster 6**
Number of patients, n (%)	1225 (20.3)	648 (10.7)	902 (14.9)	2293 (38.1)	682 (11.3)	286 (4.7)
Age, median (IQR)	77 (67-85)	75 (59-83)	70 (59-79)	63 (49-78)	72 (61-82)	82 (74-86)
Disease group						
Autoimmune (%)	2.1	1.2	1.7	1.8	2.0	1.0
Metabolic & endocrine (%)	0.0	**19.3**	**12.7**	**9.6**	**100.0**	8.0
Respiratory (%)	**9.9**	8.6	**100.0**	0.0	10.1	**15.7**
Cardiovascular (%)	**100.0**	0.0	**15.0**	0.0	**69.1**	**100.0**
Mental & neurological (%)	0.0	**100.0**	5.5	0.0	4.4	**100.0**
Neoplasms (%)	4.2	2.5	4.3	**5.9**	2.3	3.5
Digestive (%)	1.6	2.5	1.0	1.7	2.2	0.0
Genitourinary (%)	**15.6**	**9.4**	6.0	**9.0**	**15.0**	12.2
Mortality (%)	23.8	23.5	22.0	15.3	21.8	29.4

Table 5. Sensitivity analysis for the association between pre-existing health condition and in-hospital mortality in patients with COVID-19. COVID-19-related deaths were defined as COVID-19 related admissions with ICD-10 code diagnosis, including only primary or secondary diagnosis code of U07.1.

Characteristic	Level	Number of deaths, n (%)	Adjusted odds ratio (95%CI)	*P* value
Age	40-49	10 (0.8)	reference	-
	0-39	5 (0.4)	0.60 (0.20,1.77)	0.35
	50-59	59 (4.9)	3.54 (1.79,7.02)	**< 0.001**
	60-69	174 (14.4)	8.73 (4.54,16.77)	**< 0.001**
	70-79	366 (30.2)	14.24 (7.47,27.17)	**< 0.001**
	≥ 80	598 (49.3)	23.12 (12.13,44.04)	**< 0.001**
Sex	Male	704 (58.1)	1.40 (1.22,1.62)	**< 0.001**
Congestive heart failure	Yes	127 (10.5)	1.70 (1.33,2.18)	**< 0.001**
Cardiac arrhythmias	Yes	191 (15.8)	0.92 (0.76,1.12)	0.4
COPD	Yes	262 (21.6)	1.42 (1.18,1.71)	**< 0.001**
Diabetes	Yes	217 (17.9)	1.14 (0.95,1.37)	0.16
Hypertension	Yes	296 (24.4)	0.94 (0.80,1.10)	0.44
Hypothyroidism	Yes	37 (3.0)	0.91 (0.62,1.34)	0.64
Renal disease	Yes	238 (19.6)	1.84 (1.52,2.21)	**< 0.001**
Liver disease	Yes	27 (2.2)	2.46 (1.51,4.00)	**< 0.001**
Lymphoma	Yes	27 (2.2)	2.78 (1.63,4.74)	**< 0.001**
Metastatic cancer	Yes	33 (2.7)	2.18 (1.25,3.79)	**0.006**
Solid tumour without metastasis	Yes	70 (5.8)	1.69 (1.17,2.45)	**0.005**
Rheumatoid arthritis/ collagen vascular diseases	Yes	21 (1.7)	1.01 (0.61,1.69)	0.96
Obesity	Yes	21 (1.7)	5.27 (2.92,9.51)	**< 0.001**
Asthma	Yes	51 (4.2)	0.59 (0.42,0.85)	**0.004**
Depression	Yes	20 (1.7)	0.63 (0.37,1.00)	0.05
Dementia	Yes	185 (15.3)	1.43 (1.17,1.76)	**< 0.001**
Neurological disorders	Yes	72 (5.9)	1.20 (0.90,1.61)	0.22

Statistically significant $P < 0.05$ values are in bold.
Observations, n = 5955 including 1212 COVID-19 deaths
CI = Confidence Interval

Table 6. Sensitivity analysis for the association between pre-existing health conditions and in-hospital mortality in patients with COVID-19. COVID-19 deaths were defined as COVID-19 related admissions with ICD-10 diagnosis code U07.1 or U07.2 as the primary code for clinical diagnosis.

Characteristic	Level	Number of deaths, n (%)	Adjusted odds ratio (95%CI)	*P* value
Age	40-49	9 (0.8)	reference	-
	0-39	5 (0.4)	0.93 (0.31,2.82)	0.90
	50-59	51 (4.7)	3.41 (1.65,7.02)	**< 0.001**
	60-69	163 (15.0)	9.19 (4.62,18.27)	**< 0.001**
	70-79	325 (29.8)	15.21 (7.7,30.06)	**< 0.001**
	≥ 80	537 (49.3)	27.1 (13.72,53.54)	**< 0.001**
Sex	Male	633 (58.1)	1.32 (1.14,1.54)	**< 0.001**
Congestive heart failure	Yes	112 (10.3)	1.75 (1.33,2.3)	**< 0.001**
Cardiac arrhythmias	Yes	175 (16.1)	0.97 (0.78,1.19)	0.75
COPD	Yes	254 (23.3)	1.39 (1.14,1.69)	**0.001**
Diabetes	Yes	200 (18.3)	1.16 (0.96,1.41)	0.13
Hypertension	Yes	268 (24.6)	1.01 (0.85,1.2)	0.88
Hypothyroidism	Yes	32 (2.9)	0.87 (0.57,1.32)	0.50
Renal disease	Yes	220 (20.2)	1.82 (1.49,2.22)	**< 0.001**
Liver disease	Yes	19 (1.7)	2.47 (1.36,4.49)	**0.003**
Lymphoma	Yes	24 (2.2)	3.39 (1.86,6.2)	**< 0.001**
Metastatic cancer	Yes	27 (2.5)	2.11 (1.11,4.02)	**0.02**
Solid tumour without metastasis	Yes	59 (5.4)	2.08 (1.36,3.17)	**< 0.001**
Rheumatoid arthritis/ collagen vascular diseases	Yes	21 (1.9)	1.2 (0.71,2.04)	0.50
Obesity	Yes	20 (1.8)	5.06 (2.77,9.26)	**< 0.001**
Asthma	Yes	50 (4.6)	0.61 (0.42,0.88)	**0.008**
Depression	Yes	20 (1.8)	0.64 (0.39,1.05)	0.08
Dementia	Yes	169 (15.5)	1.52 (1.22,1.9)	**< 0.001**
Neurological disorders	Yes	61 (5.6)	1.45 (1.04,2.01)	**0.03**

Statistically significant $P < 0.05$ values are in bold.
Observations, n = 4960 including 1090 COVID-19 deaths
CI: Confidence Interval; COPD: Chronic obstructive pulmonary disease

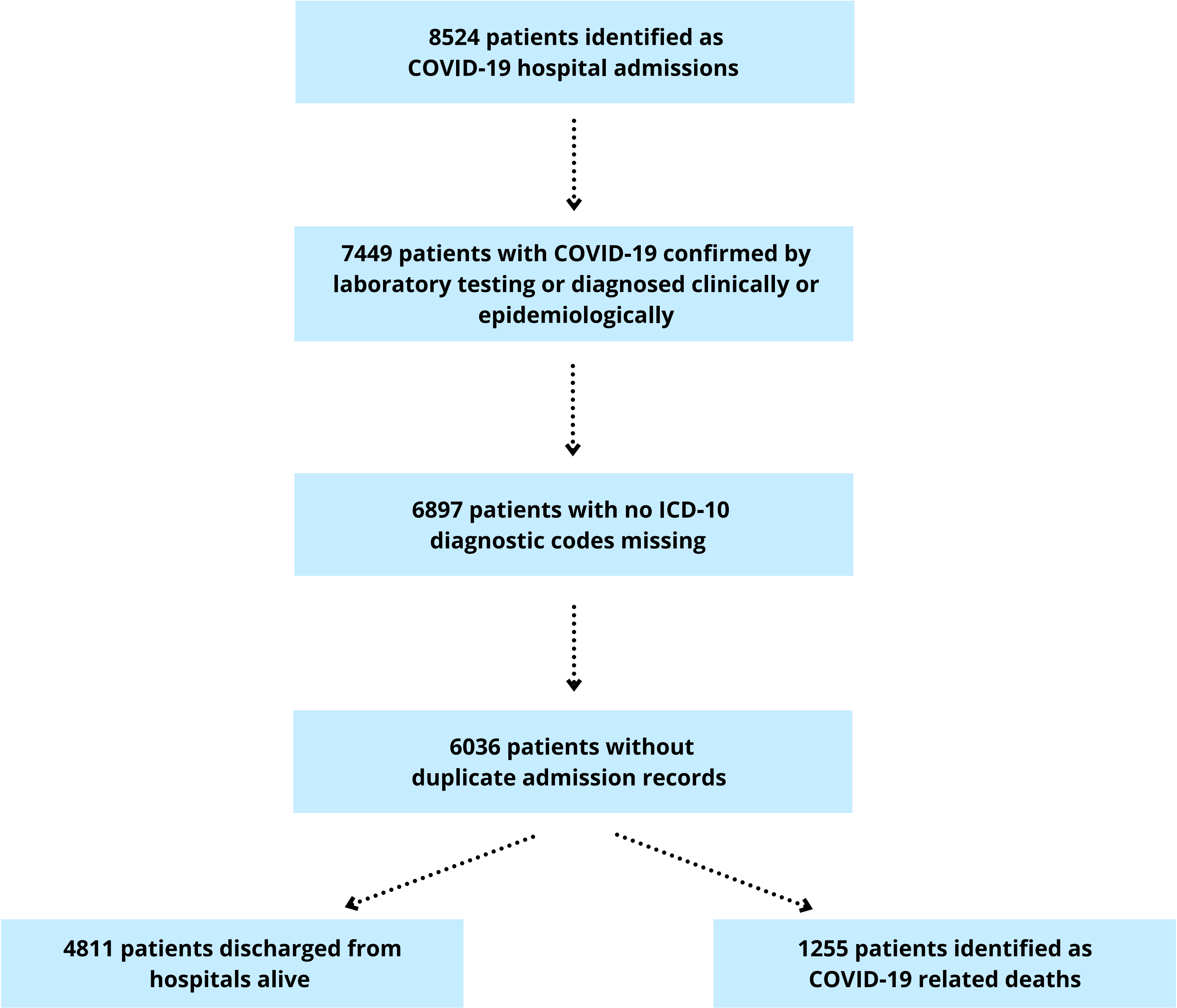
8524 patients identified as COVID-19 hospital admissions
7449 patients with COVID-19 confirmed by laboratory testing or diagnosed clinically or epidemiologically
6897 patients with no ICD-10 diagnostic codes missing
6036 patients without duplicate admission records
4811 patients discharged from hospitals alive
1255 patients identified as COVID-19 related deaths
